# Stepping Forward: A Scoping Systematic Literature Review on the Health Outcomes of Smart Sensor Technologies for Diabetic Foot Ulcers

**DOI:** 10.3390/s24062009

**Published:** 2024-03-21

**Authors:** Ioulietta Lazarou, Vasiliki Fiska, Lampros Mpaltadoros, Dimitris Tsaopoulos, Thanos G. Stavropoulos, Spiros Nikolopoulos, George E. Dafoulas, Zoe Dailiana, Alexandra Bargiota, Ioannis Kompatsiaris

**Affiliations:** 1 Information Technologies Institute, Centre for Research and Technology Hellas, 6th km Charilaou—Thermi Road, 57001 Thessaloniki, Greece; vickyfi@it.gr (V.F.); lamprosmpalt@it.gr (L.M.); d.tsaopoulo@certh.gr (D.T.); athstavr@it.gr (T.G.S.); nikolopo@it.gr (S.N.); ikom@it.gr (I.K.); 2 Institute for Bio-Economy and Agri-Technology, Centre for Research and Technology Hellas, 52124 Thessaloniki, Greece; 3 Faculty of Medicine, School of Health Sciences, University of Thessaly, 41500 Larisa, Greece; gdafoulas@uth.gr (G.E.D.); dailiana@uth.gr (Z.D.); abargio@uth.gr (A.B.)

**Keywords:** diabetic patients, diabetic foot, diabetic foot ulcers, smart insoles, sensors, actuators, scoping review, systematic literature review, digital health technologies

## Abstract

Diabetic foot ulcers (DFUs) pose a significant challenge in diabetes care, demanding advanced approaches for effective prevention and management. Smart insoles using sensor technology have emerged as promising tools to address the challenges associated with DFU and neuropathy. By recognizing the pivotal role of smart insoles in successful prevention and healthcare management, this scoping review aims to present a comprehensive overview of the existing evidence regarding DFU studies related to smart insoles, offloading sensors, and actuator technologies. This systematic review identified and critically evaluated 11 key studies exploring both sensor technologies and offloading devices in the context of DFU care through searches in CINAHL, MEDLINE, and ScienceDirect databases. Predominantly, smart insoles, mobile applications, and wearable technologies were frequently utilized for interventions and patient monitoring in diabetic foot care. Patients emphasized the importance of these technologies in facilitating care management. The pivotal role of offloading devices is underscored by the majority of the studies exhibiting increased efficient monitoring, prevention, prognosis, healing rate, and patient adherence. The findings indicate that, overall, smart insoles and digital technologies are perceived as acceptable, feasible, and beneficial in meeting the specific needs of DFU patients. By acknowledging the promising outcomes, the present scoping review suggests smart technologies can potentially redefine DFU management by emphasizing accessibility, efficacy, and patient centricity.

## 1. Introduction

Diabetes constitutes an important disease and a global health crisis that has experienced a substantial increase due to factors such as lifestyle, eating habits, and reduced physical activity [1]. Among the severe complications of diabetes, diabetic foot ulcers (DFUs) are prevalent in approximately 15% of individuals with diabetes during their lifetime [1]. DFUs present significant challenges, often requiring extended periods to heal and, in severe cases, leading to recurrent episodes, infections, and lower-limb amputations [2,3,4]. The alarming frequency of lower-limb amputations worldwide, occurring every 20 s, highlights the critical nature of diabetic foot complications and the urgent need for developing solutions that may forestall diabetic neuropathy and peripheral arteriopathy [5]. Currently, according to WHO, the annual incidence of DFU worldwide is between 9.1 to 26.1 million. On the other hand, the economic and societal impact of DFU is profound, evidenced by increased hospitalization rates and substantial healthcare costs.

Damage to the foot’s nervous and vascular systems by diabetes makes DFU formation more complicated. This leads to deformities and abnormal plantar stresses [6]. Given the imminent diabetes epidemic and the prevalent occurrence of DFUs and their recurrence, there is a clear need for improved DFU prevention and maintaining patients in remission. Current risk assessment relies on clinical and subjective evaluation, recommending therapeutic footwear for those at moderate or high risk [7]. General prevention strategies, centered around prescription footwear and orthotics, are effective when worn but often suffer from low adherence. Other traditional interventions for DFUs involve wound debridement, dressing, offloading, controlling foot infection, and managing foot ischemia [8]. Among these interventions, offloading techniques, including orthotic insoles, play a crucial role in DFU management for patients with neuropathy [9,10,11,12,13]. Studies indicate that appropriate pressure offloading significantly promotes DFU healing [14,15,16]. Meanwhile, the emergence of “smart” sensors and communication technologies like smart insoles presents new opportunities for DFU management and prevention. These technologies enable patients to monitor and input data about their feet, wounds and ulcers due to diabetes, transmitting real-time results to physicians. These cost-effective and widely accessible resources play a crucial role in predicting the risk of foot ulcers, infections, peripheral arterial disease, frailty, and other diabetes-associated complications, potentially saving limbs and lives. This complexity underlines the need for advanced approaches, such as smart insoles, to enhance the understanding and management of diabetic foot complications by monitoring plantar pressure and shear stress, which are advocated for more effective DFU management [17]. As such, early identification and intervention through technological approaches are paramount for preventing ulcer formation, given the challenges and costs associated with treating established ulcers.

So far, seven systematic reviews have shed light on the importance of sensor technology on foot plantar, but so far, none of them have examined the health dimensions of the smart insoles and devices on DFU health outcomes. The existing reviews collectively contribute valuable insights into various aspects of diabetic foot ulcer prevention, ranging from footwear design features to patient perspectives and technical descriptions of plantar pressure measurement systems. In particular, Refs. [18,19] focused on identifying the best footwear and insole design features for offloading the plantar surface to prevent foot ulceration in people with diabetic peripheral neuropathy. While it provided valuable insights into the effectiveness of certain features and pressure analysis to enhance design effectiveness, they reported no smart insole or technology-oriented footwear. On the other hand, Ref. [20] put emphasis on integrating various mechanical factors into the concept of plantar tissue stress, shedding light on the importance of considering multiple factors in diabetic foot ulcer prevention. However, the review excludes health outcomes after using smart solutions. Another systematic review by Ref. [21] explored patient and provider perspectives on smart wearable devices in DFU prevention, and despite the limited number of studies (five), the review offered only insights into the comfort, design, and usefulness of such devices. On the other hand, Ref. [22] provided a comprehensive overview of foot plantar pressure sensors and measurement systems, discussing their strengths and limitations in general, but there was no direct relevance to DFU prevention. Finally, Ref. [23] reviewed sensing technologies and discussed insights into ongoing challenges and future opportunities for contributing to the understanding of advancements in plantar pressure and stress sensing in general, whereas Ref. [24] acknowledged the multifaceted nature of DFU management and advocated a holistic approach by recognizing the potential of wearable and mobile health technologies as transformative tools in combating the alarming recurrence rates of DFU. The review explores recent advancements in technology, envisioning a future network of sensors, including skin-worn, jewelery-worn, and implantable devices. Although the review discussed how these innovations could identify high-risk patients, personalize offloading prescriptions, and enhance adherence to protective footwear, it did not apply the PRISMA guidelines and did not systematically search for studies that explored the health-related potentials linked to smart technologies for DFU.

Therefore, a critical gap in the literature is the absence of a systematic review that analyses the health outcomes of studies implementing smart sensor technologies specifically for DFUs. The present scoping review aims to fill this gap by providing a comprehensive analysis of the effectiveness of smart technologies in preventing and managing DFUs. While previous reviews offer essential building blocks, the novelty of the present review lies in its focused exploration of the health outcomes associated with smart sensor technology in DFU prevention, management, and care.

## 2. Materials and Methods

### 2.1. Review Question and Objectives

The main objective of this scoping review is to systematically map and synthesize the existing evidence on diabetic patients’ health outcomes after using smart technologies tailored for DFUs. In general, the present systematic review aims to address the overarching question: What is the effectiveness of smart sensor technologies in preventing and managing diabetic foot ulcers (DFUs)? To form the review question, the Population, Intervention, Comparison, and Outcomes (PICO) framework was adopted (see Table 1). The objectives of the review are three-fold: (1) assess the effectiveness of smart sensor technologies in preventing the occurrence of DFUs by examining studies that investigate the reduction in ulcer incidence among individuals using technological solutions compared to traditional insoles or standard care; (2) investigate the role of smart insoles in the management of existing DFUs by examining studies that explore the impact of smart insoles on ulcer healing rates, recurrence, and overall wound management; and (3) examine the level of patient adherence to smart insole usage. By systematically addressing these objectives, this review seeks to provide a comprehensive and up-to-date analysis of the effectiveness of smart insoles in both preventing and managing diabetic foot ulcers, offering insights that can inform clinical practice and guide future research on DFUs.

### 2.2. Search Strategy

**Figure 1:**
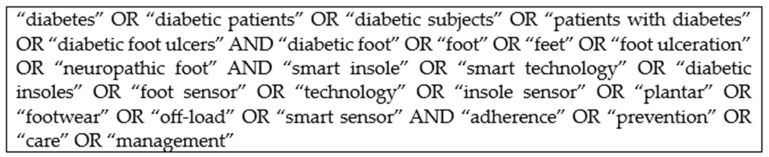
Search strategy used in MEDLINE and modified for other databases.

A systematic and thorough search strategy was employed to identify relevant studies investigating the effectiveness of smart insoles in preventing and managing DFUs. The search encompassed major electronic databases, including PubMed/MEDLINE, Scopus, and Web of Science. The search strings were carefully crafted using a combination of controlled vocabulary (MeSH terms) and pertinent keywords (see Figure 1 for the search strategy used for MEDLINE). Boolean operators were used to refine and broaden the search. The inclusion criteria focused on original research articles, clinical trials, and observational studies published in English from the inception of the databases to the present date. Manual hand-searching of reference lists from the included studies was added, while systematic reviews and meta-analyses were also considered but were not included in the final studies. The search strategy aimed to be comprehensive, ensuring that a diverse range of studies was considered to provide a robust foundation for this systematic review’s objectives. The specific years of analysis were selected based on the availability of the literature relevant to the topic of interest. Our aim was to capture the most recent and comprehensive evidence pertaining to the use of smart technologies in the management of diabetic foot ulcers (DFUs), particularly considering that smart technologies are relatively recent developments in healthcare, with significant advancements occurring after 2013. By focusing on studies published within a defined timeframe, typically from 2013 onwards, we aimed to ensure that our review reflects the latest developments and advancements in this rapidly evolving field. Additionally, restricting the search to a specific timeframe helps to manage the scope of the review and ensures that the included studies are contemporary and relevant to current clinical practice.

### 2.3. Eligibility Criteria

Articles were included if they met all the following a priori specified criteria:

- Primary research studies (e.g., pilot, RCTs, etc.);
- Full-text research articles;
- English-language publications;
- Studies conducted with adult (>18) patients with diabetes and DFU with a history of neuropathy/foot ulcerations;
- Studies reporting the use of sensor health technologies, such as smart insoles;
- Studies reporting patients’ health outcomes after using the suggested technology (e.g., smart insoles).

Papers were excluded if they were reporting on the following:

- Non-primary studies, including systematic reviews;
- Opinion articles, editorials, and book chapters;
- Non-English-language publications;
- Studies with non-adult (<18) diabetic patients;
- Studies reporting the use of clinician-, caregiver-based smart insoles;
- Studies solely exploring technological advances of a developed smart solution;
- Studies exploring smart insoles for other purposes and not diabetic foot (e.g., gait);
- Studies exploring other health-related factors related to diabetes (e.g., glucose);
- Other medical conditions not related to diabetes (e.g., foot wounds due to other syndromes);
- Technologies using only smartphones/apps for detecting foot ulcers or telemedicine using advocacy;
- Testing of tests and devices available on the market but not technological ones (e.g., Neuropad);
- Studies reporting clinicians’/caregivers’ perceptions on the use of the suggested technologies.

### 2.4. Selection Procedure

The process of selecting studies comprised three distinct phases. Initially, all records obtained through database searches underwent a review based on their titles to assess eligibility. Subsequently, a selection procedure was carried out considering abstract information. Finally, studies meeting the inclusion criteria from the initial two phases underwent a comprehensive full-text review. The eligibility of obtained records was independently assessed by three reviewers (IL, VF, and LM). Any disparities encountered at each stage of the study selection process were resolved through discussion. Additionally, citation chaining, both forward and backward, was employed to guarantee the identification and inclusion of all relevant publications deemed eligible.

### 2.5. Data Extraction

**Figure 2:**
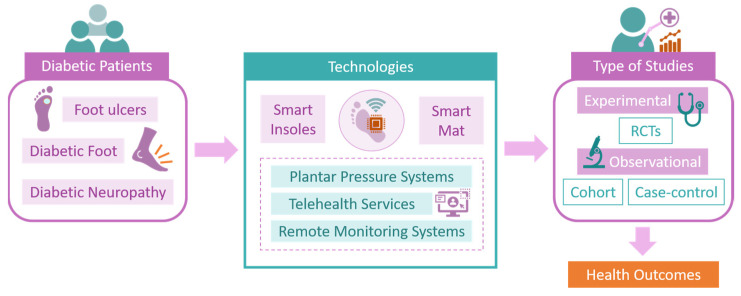
Data extraction results overview for the systematic review.

Independently, the three reviewers systematically gathered pertinent data from each included study, documenting the technologies used and participants’ health-related outcomes after using the suggested technologies. Independent reviewers conducted the initial screening of titles and abstracts to identify potentially relevant articles. Full-text articles were then assessed for eligibility based on the inclusion and exclusion criteria. This process involved the use of a standardized form specifically developed for this review. For each study, the following details were extracted and recorded in the form: Author(s), publication date, study design, study population, sample size, type of technology used, intervention, and the health outcome. The outcomes of the data extraction were cross-referenced, and any discrepancies among reviewers were resolved through consensus. As such, relevant data, including study design, sample size, sensor technology employed, diagnostic outcomes, and key findings, were systematically extracted from the selected studies. Figure 2 outlines the review pipeline for the data extraction and results for the purposes of the present review.

**Figure 3:**
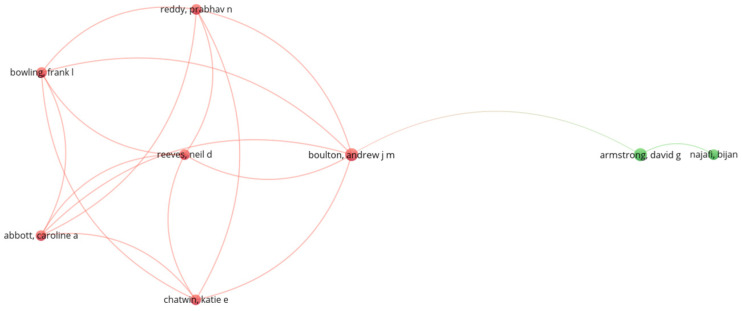
Network visualization (The color of the circle of an item determining the cluster to which the item belongs).

The resulting 11 scientific publications (Table 2) were subjected to analysis through VOSViewer (https://www.vosviewer.com/, 4 March 2024), a bibliometric tool used for constructing and visualizing networks, insights into the structure, and interconnections within the literature corpus of the presented scoping review. The resultant network visualization (Figure 3) depicts nodes representing individual publications, with edges denoting the relationships between them. Through the examination of node proximity and clustering, thematic similarities and connections among studies become apparent, providing valuable insights into overarching themes and trends prevalent in the research domain.

**Figure 4:**
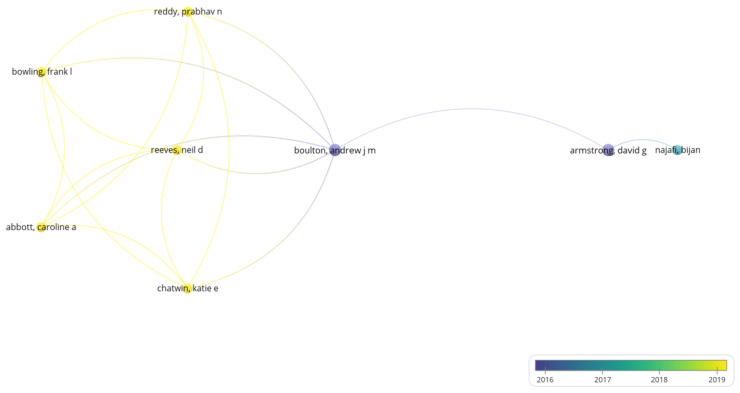
Overlay visualization.

Overlay visualization (Figure 4) within VOSviewer facilitates the integration of supplementary metadata or attributes linked to each publication, such as publication year. This augmentation discerns temporal patterns and trends, identifying seminal journals or prolific authors contributing to the field and scrutinizing the dissemination of concepts throughout the literature. Consequently, this approach fosters a nuanced understanding of the evolutionary trajectory of research pertaining to smart sensor technologies for diabetic foot ulcers, thereby furnishing pertinent insights into key contributors and salient topics within the domain.

**Figure 5:**
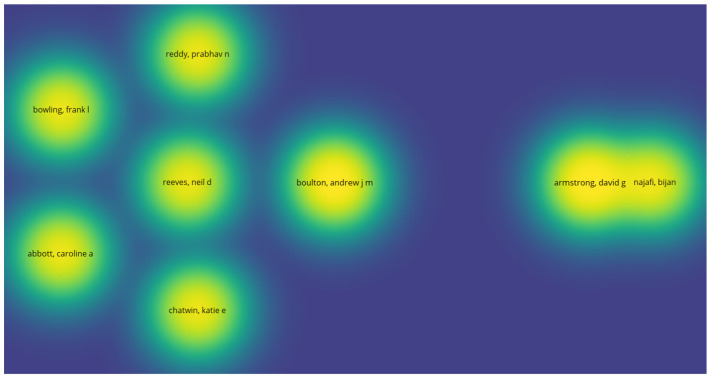
Density visualization.

Moreover, density visualization (Figure 5) within VOSviewer affords a quantitative appraisal of the concentration and density of connections inherent within the network. By quantifying the density of connections between publications within delineated network regions, areas of heightened research activity or thematic focus are accentuated. The visualization of density aids in pinpointing clusters of closely interconnected publications, indicative of areas characterized by concentrated research efforts or trends within the literature corpus. This analytical technique serves as a mechanism for identifying research lacunae, delineating avenues for further exploration, and fostering prospects for collaborative ventures and interdisciplinary inquiry within the realm of smart sensor technologies for diabetic foot ulcers and their attendant health outcomes.

## 3. Results

### 3.1. Search Results

**Figure 6:**
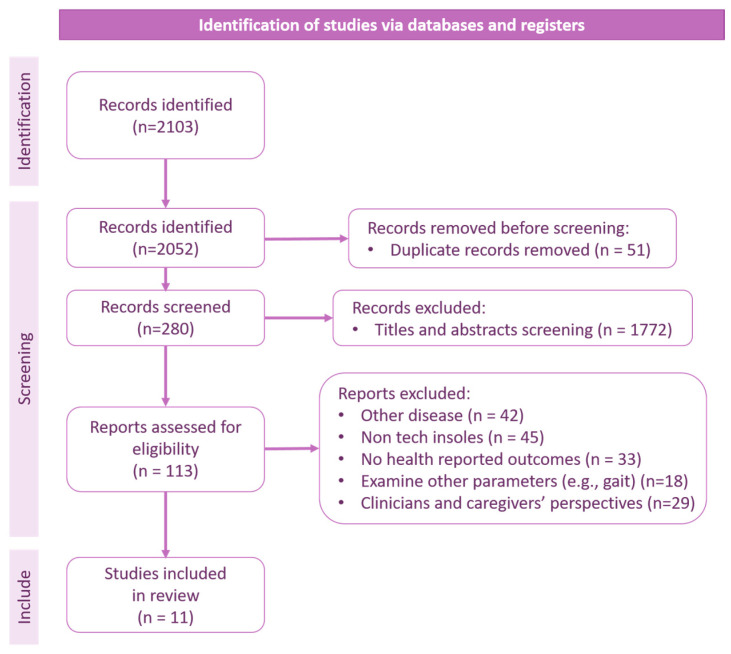
PRISMA flowchart.

Database searches yielded a total of 2103 records. After duplicates were removed, 2052 studies were screened based on titles, and 280 studies were screened on the basis of abstracts. Following the screening of titles and abstracts, 113 potentially eligible articles remained, which were examined in full. Of these studies, 11 met the criteria for inclusion (see Figure 6 for the PRISMA flow diagram of the study selection process [25]).

### 3.2. Description of Included Studies

Overall, the included studies were published within the last 18 years (2005–2023), with the overwhelming majority (n = 10) being published from 2017 onwards. Studies employed different research designs, with most (n = 5) being interventional or pilot randomized controlled trials (RCTs) or small-scale observational investigations. Data collection in all studies was conducted prospectively. Sample size varied greatly between studies (from 5 to 174 participants), reflecting the diverse objectives and research designs employed by the included studies. The participant population consisted of diabetic patients with diabetic foot ulcerations or a history of neuropathy, while some had also employed a control group for cross-sectional analysis. Table 2 presents an overview of the characteristics of the included studies. The outcomes, methodologies, and implications of these studies were examined to provide a deep understanding of the diverse approaches in the DFUs. In the broader context, the studies collectively highlighted the effectiveness of intelligent insole systems [26,27], adherence to alert-based cues [28], in-shoe plantar pressure analysis [29], real-world impact [30], health coaching [31], wearable sensor-based insoles [32,33], smart mats [34,35], and smart socks [36]. Each intervention brings unique strengths, and while some studies focus on specific technologies, others explore comprehensive approaches, including health coaching and therapeutic footwear modifications.

**Table 2:** Study characteristics.

Author(s), Year	Study Design	Study Population and Size	Technology	Intervention	Health Outcomes
[33]	RCT	N = 50 DFU	Smart insoles (removable cast walker (RCW)) and an “instant” total contact cast (iTCC)	A 12-week evaluation or until wound healing; Group 1: removable cast walker (RCW); Group 2: “instant” total contact cast (iTCC).	Higher proportion of patients had healed ulcers in the iTCC group than in the RCW group; Those treated with an iTCC healed significantly sooner (82.6 vs. 51.9%, *p* < 0.02, odds ratio 1.8 [95% CI 1.1–2.9]); Of the patients with ulcers that healed, those treated with an iTCC healed significantly sooner (41.6 ± 18.7 vs. 58.0 ± 15.2 days, *p* = 0.02).
[29]	Cross-sectional study	N = 23 DPU with neuropathy	Smart Insole Sensor	A 12 m walkway while in-shoe plantar pressures were measured in four walking trials.	Effective and efficient in-shoe plantar pressure analysis to evaluate and guide footwear modifications that significantly reduce pressure in the neuropathic diabetic foot; In 35 defined regions, 30.2% pressure relief (range 18–50% across regions).
[28]	Prospective cohort study	N = 17 DFU with neuropathy	Smart Insole Sensors and Actuators (SurroSense Rx by Orpyx Medical Technologies Inc., Calgary, AB, Canada)	A 3-month period to cue for unprotected sustained plantar pressures	Patients demonstrating increased adherence over the course of this study received more alerts; Participants who had received at least one alert every two hours were more adherent with offloading than participants who received fewer alerts (52.5 ± 4.1% vs. 24.7 ± 22.4%, *p* = 0.043).
[34]	Prospective, multicenter, cohort study	N = 132 DFU	Smart mat (podimetrics mat)	Follow-up at 34 weeks	The system correctly identified 97% of observed DFU; A total of 86% of the cohort used the system at least 3 days a week on average over this study.
[36]	Pilot study	N = 33 DFU with neuropathy	Smart insole-socks (SmartSox, Rochester, NY, USA)	Habitual gait-speed in a clinical setting Estimation of temperature, pressure, and toe range of motion	Validity of an innovative smart textile for assessing simultaneously the key parameters associated with risk of foot ulcers; Empower clinicians to objectively stratify foot risk and provide timely care; A significant correlation was found for pressure profile under different anatomical regions of interest between SmartSox and F-Scan (r = 0.67, *p* < 0.050), as well as between thermography and SmartSox (r = 0.55, *p* < 0.050).
[30]	Case study	N = 1 DFU and severe peripheral neuropathy	Smart insole system	One month monitoring	No significant differences in pressure were present for the right foot with the embedded screw; The contralateral foot showed significantly higher pressure when the screw was embedded; The number of bouts of high pressure per hour showed a significant increase in the left foot (*p* < 0.001) during the time the screw was embedded in the shoe.
[27]	Mixed method (Prospective, RCT proof-of-concept)	N = 90 with diabetes	Smart insole, sensors, and smartwatches	A total of 18 months of recording/feedback via audio-visual-vibration alerts; IG-received audiovisual alerts via a smartwatch; CG did not receive any alerts.	At follow-up, ten ulcers were recorded in the CG and four ulcers in the IG; A 71% reduction in ulcer incidence in the IG compared with the CG.
[35]	Cohort study	N = 129 with diabetes and previously healed DFU	Smart mat (Podimetrics RTM System by Podimetrics Inc., Somerville, MA, USA)	Once-daily foot temperature monitoring for predicting foot ulceration for 34 weeks	Telemedicine mat is no less accurate in predicting the development of DFU in patients with recent wounds and in patients with partial foot amputations than in those without these potentially challenging conditions; Non-inferior predictive accuracy in each of the two potentially challenging cohorts relative to the control cohort (α < 0.05). The alert lead time was similar across these cohorts, ranging from 33 to 42 days.
[26]	Longitudinal RCT study	N = 46 DFU	Smart insole, sensors, and smartwatches	All-day use for 18 months, recording and providing pressure feedback via audio-visual-vibration alerts Patients were allocated to IG and CG.	Controls experience more high-pressure bouts than the intervention group Differences between groups apparent at >16 weeks CG experienced more high-pressure bouts over time than IG across all areas of the foot (*p* < 0.05).
[31]	Mixed-methods intervention	N = 10 with diabetes and related peripheral neuropathy	Health coaching training and smart insole sensors and actuators (SurroSense Rx by Orpyx Medical Technologies Inc.)	A 4-week explanatory sequential mixed-methods intervention	Improvement in participant understanding of neuropathy, foot care behaviors, and intention to adopt the smart insole; Mean smart-insole wear was 12.53 ± 3.46 h/day, with 71.2 ± 13.9% alerts not effectively offloaded and with no significant effect for time on usage: F(3,6) = 1.194 (*p* = 0.389) or alert responses F(3,6) = 0.272 (*p* = 0.843).
[32]	Cross-sectional study	N = 6 (5 DFU and 1 healthy participant)	Smart insoles	Laboratory evaluation performs level walking along a 28 m corridor at a self-selected speed	The performance of the sensorized insole system is comparable to previously reported research devices; Adequate sensitivity and safety to assist footwear assessment relevant to foot ulcer prevention for people with diabetes; Change in footwear resulted in approximately 20%, 75%, and 82% change in pressure, medial–lateral and anterior–posterior shear stress, respectively.

### 3.3. Description of Identified Digital Health Technologies

All the final included studies highlighted the potential of intelligent insole systems in preventing and managing DFUs. The continuous monitoring of plantar pressure, coupled with personalized feedback, demonstrated a reduction in high pressure and, consequently, a decline in DFU incidence. All studies investigated various interventions, ranging from intelligent insole systems to remote foot-temperature monitoring and health coaching, aiming to prevent and manage DFUs.

#### 3.3.1. Intelligent Insole Systems: Continuous Monitoring and Feedback

Studies by [26,27] focused on intelligent insole systems for preventing DFUs. In particular, in the [26] study, an 18-month RCT involving 46 patients with diabetic peripheral neuropathy and previous DFU, an intelligent insole system was used for continuous plantar pressure monitoring. The intervention group (IG) received audio-visual-vibrational alerts for high-pressure bouts, while the control group (CG) did not receive any feedback. CG experienced more high-pressure bouts over time across all foot areas, with significant differences emerging after 16 weeks; however, the IG showed a learning response, pre-emptively offloading to avoid alerts, resulting in a reduction in high-pressure bouts. Their results showed that the intelligent insole system, providing personalized feedback, demonstrated a 71% reduction in DFU incidence, highlighting the potential for continuous pressure feedback in high-risk patients. In this common vein, Ref. [27] conducted a prospective, randomized proof-of-concept study aimed to reduce DFU recurrence in 90 high-risk patients using an intelligent insole system. All participants were assigned to IG or CG, and both received insole systems for continuous plantar pressure monitoring, but the IG also received audiovisual alerts for aberrant pressures. This study reported a remarkable 71% reduction in ulcer incidence in the IG compared with the CG, emphasizing the effectiveness of dynamic offloading guidance. Individual plantar sites ulcerated less frequently in the IG, and exploratory analysis revealed an 86% reduction in ulcer incidence among good compliers in the IG. Both share similarities in their methodology, including the use of intelligent insole systems, continuous plantar pressure monitoring, and a control group without pressure feedback. While Ref. [26] focused on reducing high-pressure bouts in high-risk patients, Ref. [27] targeted the prevention of DFU recurrence. Both studies emphasize the importance of individualized, dynamic offloading guidance through audiovisual alerts. However, in Ref. [26], patients reported a learning response after approximately four months, while in Ref. [27], they demonstrated a substantial reduction in ulcer incidence within the 18-month follow-up, which underlines the scope of intelligent insole systems beyond initial prevention, addressing a critical aspect of diabetes-related foot complications.

Two studies also tested a similar sensor. In particular, a prior case study by Ref. [30] with a 59-year-old patient with type 2 diabetes and severe peripheral neuropathy tested SurroSense Rx for monitoring and preventing potential foot complications in individuals with diabetic neuropathy. Data analysis revealed that despite the embedded screw, there were no significant differences in pressure analysis for the right insert. However, the left insert showed a significant increase in the total minutes of high pressure per hour and the number of bouts of high pressure per hour during the period when the screw was embedded in the shoe. This finding indicated that the contralateral foot experienced an increase in pressure, potentially elevating the risk of ulceration during this period. The case study highlighted the importance of the plantar pressure feedback system in capturing the effects of a foreign object in the shoe, demonstrating its potential to detect changes in plantar pressures in real-world scenarios. Notably, the participant’s severe neuropathy prevented him from feeling the embedded screw, emphasizing the system’s role in providing crucial feedback in the absence of sensory perception. The study suggested that the innovative SurroSense Rx system, designed for continuous pressure analysis and feedback in daily life, could be instrumental in preventing foot complications in individuals with diabetes and neuropathy. In this common vein, Ref. [28] conducted a comprehensive study evaluating patient adherence to a pressure-sensitive insole system (SurroSense Rx) designed to assess plantar pressures and provide alert-based feedback in individuals at high risk of DFU. The study revealed promising results, indicating that patients found the technology acceptable and beneficial. Adherence was examined in the context of the number of daily alerts, attempting to understand its impact on daytime device adherence. Despite the encouraging findings, the study’s sample size (n = 12) was acknowledged as a limitation, prompting the need for a larger-scale study to validate observations. Moreover, the study acknowledged limitations related to the device’s inability to collect data during non-wearing periods, emphasizing the importance of understanding true adherence to footwear during various foot-loading conditions. As a result, the study suggested that the proposed alert-based device had the potential to enhance adherence to prescribed footwear and might reduce the risk of ulcer recurrence in high-risk populations. The improved adherence observed in the study’s high-risk group indicated that the alert-based device could play a crucial role in preventing recurrent plantar ulcers. As such, the study provided valuable insights into the acceptance and perceived benefits of smart insoles with alert-based feedback among high-risk diabetic patients.

Another research approach by [29] focused on therapeutic footwear for diabetic foot patients, aiming to reduce ulceration risk by relieving mechanical pressure on the foot. Dynamic in-shoe plantar pressure distribution was measured, and footwear modifications were made to optimize pressure reduction. The study demonstrated a 30.2% pressure relief across regions, suggesting that in-shoe plantar pressure analysis is an effective and efficient tool to guide footwear modifications for pressure reduction in neuropathic diabetic feet. The Pedar-X system, a flexible pressure-sensing insole with 99 sensors, was calibrated, patients walked along a 12-m walkway, and in-shoe plantar pressures were measured during the walking trials. The optimized footwear was associated with reduced pressure at targeted regions, suggesting a potential reduction in the risk of DFU. The results indicated that positive outcomes could be achieved by experienced clinical teams, emphasizing the potential of the optimization approach to provide better quality footwear for individual patients. Despite the study’s limitations, including the small sample size and the absence of objective criteria for modifications, the study concluded that in-shoe plantar pressure analysis offered an effective and efficient method for assessing and improving therapeutic footwear quality, potentially reducing the risk of plantar foot ulceration in neuropathic diabetic foot patients.

#### 3.3.2. Remote Foot-Temperature Monitoring

In the study conducted by [34], a prospective and multicenter cohort approach was employed to assess the effectiveness of a novel remote foot-temperature monitoring system using a wireless thermometric foot mat. With a focus on 132 diabetic participants with a history of healed DFUs, the study aimed to evaluate the device’s accuracy in predicting nontraumatic plantar DFUs. The 34-week follow-up period included participants from various care environments, while the study device, known as the Remote Temperature Monitoring System, featured a wireless floor mat with temperature sensors, facilitating daily foot temperature scans. The device demonstrated a high accuracy of 97% in detecting DFUs at a temperature asymmetry threshold of 2.22 °C. Despite concerns about in-home monitoring adherence, the study reported encouraging compliance, with 86% of participants averaging at least three uses per week. However, several losses to follow-up were observed, suggesting varied adoption rates among participants. As such, the findings underscore the importance of technological interventions in enhancing adherence and early intervention for improved diabetic foot care. In this common line, the study by [35] focused on assessing the accuracy of once-daily foot temperature monitoring for predicting foot ulceration in diabetic patients, specifically those with recent wounds and partial foot amputations, using a telemedicine mat, the Podimetrics RTM System. The study analyzed data from 129 participants with previously healed diabetic foot ulcers. Among the most important findings, non-inferior predictive accuracy in cohorts with recent wounds and partial foot amputations compared to the control cohort showcased the reliability of once-daily foot temperature monitoring in challenging conditions. The study reported a consistent alert lead time ranging from 33 to 42 days across cohorts, emphasizing the potential for early detection. Participants were followed for 34 weeks, and the once-daily foot temperature monitoring, facilitated by the telemedicine mat, demonstrated accuracy in predicting foot ulceration, while another advantage of this approach was that the mat’s large surface area accommodated patients with foot deformities or amputations, contributing to its practicality.

#### 3.3.3. Health Coaching and Wearables

A more recent approach by [31] evaluated the feasibility of podiatrist-led health coaching to facilitate smart insole adoption and foot monitoring in adults with diabetes-related neuropathy. The study employed a quantitative dominant mixed-methods intervention with an explanatory sequential core design in a 5-week intervention period, and the data collection tools included a health coaching fidelity assessment tool, SurroSense Rx* smart insoles for monitoring, and questionnaires. The health coaching training package was delivered to participating podiatrists, emphasizing individualized foot health monitoring goals and the use of smart insoles. Participants reported improved foot care behaviors. However, the qualitative data revealed challenges, including frustration with device malfunctions, perceived burdens, and disruptions caused by audible alarms. Podiatrists’ health coaching fidelity scores indicated a need for further reinforcement of coaching skills. Participants reported that health coaching enhanced their understanding of foot health but expressed frustration with device elements and disruptions caused by alarms during social activities. The results indicated that health coaching positively influenced communication and understanding but did not fully address participant dissatisfaction with some aspects of smart insole usage.

On the other hand, Ref. [33] conducted a study with the objective of evaluating the effectiveness of a removable cast walker (RCW) and an “instant” total contact cast (iTCC) in healing 50 neuropathic DFUs. The iTCC group showed a higher proportion of healed ulcers at 12 weeks compared with the RCW group, with those treated with iTCC healing significantly sooner. The modification of the standard RCW to enhance patient adherence to pressure offloading proved to increase the proportion of healed ulcers and the rate of healing in diabetic neuropathic wounds. The study emphasizes the importance of patient adherence and suggests that modifications to offloading devices can significantly impact the outcome of wound healing in DFUs. Additionally, the study discusses the challenges posed by the technical difficulty and time consumption, making them less widely used despite their effectiveness, and highlights the potential of modified RCWs, such as iTCC, as a more practical and effective alternative in clinical practice. In this common line, the study by [32] focused on the design, development, and evaluation of a sensorized insole system incorporating TRIPS sensing technology. The TRIPS sensors use a capacitive sensing mechanism to measure pressure and shear stresses simultaneously. The insole, comprised of three layers (EVA, synthetic leather, and Lycra), integrates these sensors to measure pressure and shear across different plantar sites in real time. Laboratory-based and human participant tests, including a healthy male participant walking in various footwear conditions, were conducted to assess the insole’s performance. The study reported that the smart insole, designed for use without footwear modification, presented a significant advancement in usability for everyday DFU prevention, offering insights into biomechanical aspects and loading characteristics in real-world settings.

All studies showed and emphasized the importance of personalized, continuous feedback in DFU prevention. In particular, they demonstrated that continuous plantar pressure monitoring, coupled with dynamic offloading guidance, significantly reduces DFU site recurrence, underscoring the potential of intelligent insole systems in clinical settings. The potential of smart insoles, coupled with alert-based feedback, to enhance adherence to prescribed footwear was highlighted in the majority of the reported studies, indicating further exploration in larger sample sizes and extended follow-up periods. Adherence to continuous monitoring systems varied, which shows that interventions may depend on patient engagement. In particular, health coaching interventions, while proving to be feasible, require further daily assistance. Additionally, the studies have varying follow-up durations, ranging from 3 months to 18 months, influencing the understanding of long-term outcomes of smart devices on preventing DFUs. Continuous monitoring, early detection, and patient engagement remain pivotal in preventing diabetic foot ulcers. As the research around smart insoles for DFU care advances, the integration of all the knowledge gained from the abovementioned research approaches and the incorporation of patient perspectives will be crucial for advancing diabetic foot care strategies.

## 4. Discussion

The present scoping review aimed to identify and synthesize existing evidence on available smart sensor technologies for DFU management, care, and prevention. The integration of sensor systems and offloading devices emerges as a pivotal solution in the management of the DFUs. The increase in technological advancements and clinical interventions reflects a concerted effort to address the multiple challenges associated with ulcers as a result of diabetes, ranging from early detection to effective offloading strategies, health coaching, and prevention or forestalling of the disease. Our review findings show that several research studies that have extensively investigated the diabetic population with a history of foot ulcerations or the potential advantages of using such systems for DFU care.

The studies present a clinical-meaningful contribution to the DFU by introducing sensor insole systems equipped with smart technology for DFU management. The real-time monitoring of pressure and shear stresses across different plantar sites offers unparalleled insights into DFU management. This approach not only enhances the understanding of balance and impulses but also provides a comprehensive assessment beyond traditional pressure measurements. A notable strength of [32] smart insole lies in its integration into daily living environments without necessitating footwear modifications. The unobtrusive nature of the wearable system suggests it is a potential early detecting tool for patients and healthcare professionals, focusing on elevated DFU risks promptly. The study’s innovative approach to combining pressure and shear assessment highlights the inadequacy of relying solely on pressure metrics for a comprehensive understanding of loading characteristics. However, the translation of sensor technologies from controlled laboratory settings to real-world clinical utility requires further examination. While the abovementioned research work of [32] offers promising initial results, the sample size of healthy participants and the absence of a diverse patient population limit the generalizability of the findings. Moreover, the complexity of sensor technologies may drive challenges in terms of cost-effectiveness and accessibility, especially in healthcare settings. On the other hand, Ref. [31] showed valuable insights into the integration of telehealth and sensor technologies. The incorporation of wearable sensors in a telehealth platform allows for remote monitoring, suggesting early intervention and personalized care. Moreover, the results from [24] showed the potential of sensor systems in diabetic foot care by exploring the utility of in-shoe plantar pressure monitoring. The study, focused on patients with diabetic neuropathy, elucidates the relationship between plantar pressure and ulceration risk. The findings indicated the importance of continuous monitoring in high-risk populations, offering a proactive means for preventing DFUs through the identification of pressure-induced vulnerabilities.

Studies by [27,29,33,34] shed light on the efficacy of offloading devices in DFU management. In particular, by comparing RCWs and iTCCs, the study [33] revealed that the modification of standard offloading devices can significantly enhance patient adherence and, consequently, ulcer healing rates. The efficacy of offloading devices, such as the iTCC, in expediting wound closure not only demonstrates a higher proportion of healed ulcers in the iTCC group but also underlines the importance of time to closure as a critical metric. The present findings suggest that modifying existing offloading devices can bridge the gap between traditional total contact casts and more user-friendly alternatives, potentially improving their adoption and efficacy in clinical practice. Moreover, the study [34] exploring the TCC, a well-established but technically demanding offloading device, reaffirms its efficacy in healing neuropathic ulcers. The study also highlights the challenges associated with the widespread adoption of TCCs due to their technical intricacy and time-consuming application. Moreover, Refs. [27,29] further highlight the complexities of offloading interventions. The investigation into custom-made footwear emphasizes the role of personalized solutions in managing plantar pressure, reducing ulcer recurrence, and improving patient outcomes. The tailored approach aligns with the concept of precision medicine, recognizing the heterogeneity of DFU presentations and the need for personalized interventions. However, the potential drawbacks of offloading devices, particularly in terms of patient adherence, include problems associated with patients easily removing devices.

The findings from sensor systems and offloading devices underscore the urgent need for an integrated and patient-centric approach to DFU management. While sensor technologies offer real-time insights and early signs for clinicians, offloading devices contribute to the actualization of preventive strategies and wound healing. A combined solution to these approaches might have the potential to improve DFU care by addressing the spectrum of challenges faced by both clinicians and patients with DFU. The prospect of integrating sensor systems with telehealth platforms, as explored by [31], represents a stepping stone toward giving access to specialized diabetic foot care. The remote monitoring capabilities afforded by wearable sensors align with the broader trend of telemedicine, ensuring continuous surveillance and timely interventions. As such, the evolving landscape of DFU management is marked by the convergence of technological innovation and clinical-related daily problems. The studies discussed herein collectively contribute to our understanding of the strengths and limitations of sensor systems and offloading devices. The studies laid the foundation for future research, emphasizing the need for larger, randomized controlled studies to clinically validate the effectiveness of smart sensor technologies in preventing the recurrence of plantar ulcers.

In addressing the global burden of diabetic foot disease, long-term medical management aims to reduce DFU risk, and recurrence is of high importance. The pervasive integration of technology into various facets of daily life presents a unique avenue for innovative solutions in the prevention and management of diabetic foot problems. Recent studies in wearable health technologies, in particular, hold promise for quantifying and regulating foot pressure and inflammation, thereby extending periods of remission and enhancing the quality of life for patients with diabetes and DFU health challenges. The advent of smart sensors and communication technologies has introduced opportunities to intelligently address DFUs through personalized screening and timely interventions. By leveraging automation, novel solutions are emerging to deliver comprehensive and user-friendly feedback, personalized guidelines, and timely notifications. For instance, incorporating health coaching techniques can engage patients and enhance adherence to offloading recommendations. These technologies serve as supportive tools, empowering patients in self-care by facilitating routine measurements and prompt feedback on inflammatory responses, plantar stress variations, and daily activities. By transmitting real-time results to healthcare providers, these cost-effective devices prove to be instrumental in predicting an individual’s susceptibility to foot ulcers, infections, peripheral arterial disease, frailty, and other complications associated with diabetes. In essence, these technologies not only contribute to limb preservation but also play a crucial role in saving lives.

While smart sensor technologies offer promising solutions for the monitoring and diagnosis of diabetes mellitus and DFU, they also present certain challenges that need to be addressed. One of the primary challenges is the integration of diverse data streams generated by these technologies into a cohesive and user-friendly platform. With the proliferation of wearable devices, remote monitoring systems, and mobile health apps, there is a risk of data overload and fragmentation, making it difficult for healthcare providers to interpret and use the information effectively. Furthermore, ensuring the accuracy and reliability of data collected from these technologies remains a critical concern. Variability in sensor accuracy, data interoperability issues, and the potential for technical malfunctions can compromise the quality of the data obtained, leading to erroneous interpretations and clinical decisions. Additionally, the lack of standardization in data collection protocols and device interfaces poses challenges for seamless integration into existing healthcare workflows. Another significant challenge is the accessibility and affordability of modern technologies, particularly for underserved populations and resource-constrained settings. While advancements in technology have led to the development of innovative monitoring solutions, disparities in access to these technologies persist, hindering their widespread adoption and impact. Moreover, ethical considerations surrounding data privacy, security, and informed consent need to be carefully addressed to safeguard patient confidentiality and autonomy. The collection, storage, and sharing of sensitive health information via digital platforms raise concerns about the potential misuse of personal data. Addressing these challenges requires collaborative efforts from healthcare providers, technology developers, policymakers, and regulatory bodies. Establishing robust quality assurance mechanisms, promoting interoperability standards, and fostering inclusive design approaches are essential steps toward harnessing the full potential of modern technologies for diabetes management.

As such, these studies provide compelling evidence for the effectiveness of intelligent insole systems in preventing DFUs and reducing recurrence. Further research and technological advancements in this field hold the potential to revolutionize the management of DFUs, improving the quality of life for individuals with diabetes. In conclusion, these 11 studies collectively contribute valuable insights into DFU prevention and management. Intelligent insole systems, remote monitoring technologies, health coaching, and wearable sensorized insoles show diverse approaches, each with unique strengths and considerations. The findings underline the potential for personalized, continuous monitoring and feedback to revolutionize diabetic foot care. Further research, refinement of interventions, and technological advancements hold the key to improving outcomes for individuals with diabetes at risk of foot ulcers. While each study addresses specific aspects of DFU prevention and management, several commonalities and diverse approaches emerge. Intelligent insole systems, continuous monitoring, and personalized feedback demonstrate consistent efficacy in reducing DFU incidence and recurrence. The importance of patient adherence to alerts and the potential of in-shoe plantar pressure analysis in guiding footwear modifications proved to be of high importance, while remote monitoring technologies, such as foot-temperature monitoring, present promising accuracy for predicting DFUs. Moving forward, a concerted effort is needed to bridge the translational gap, refining sensor technologies for real-world applicability and enhancing the accessibility and user-friendliness of offloading interventions. Collaborative initiatives between researchers, clinicians, and technology developers are pivotal in shaping the future of DFU care, fostering a holistic and personalized approach that transcends the traditional boundaries of diabetic foot management.

## 5. Conclusions

The present systematic scoping review marks a pioneering effort in comprehensively assessing the impact of smart technologies on the health outcomes of DFU patients. The results reveal a field characterized by promising advancements in sensor systems and offloading devices, offering a glimpse into the future of DFU management. Among the technologies, three distinct categories emerge: smart insoles for pressure monitoring and adjustment, smartwatches with coaching functionalities, and temperature monitoring devices. Data extraction indicates that smart insoles hold considerable promise, offering a means to actively monitor and potentially alter pressure distribution, thereby reducing the risk of ulcer development or progression. Additionally, temperature monitoring emerges as a potentially accessible and cost-effective tool, either as a standalone intervention or as a complementary technology alongside more sophisticated sensor systems. Furthermore, our review underscores the potential of self-management and patient observation in fostering awareness and behavior change. Empowering patients with tools for self-monitoring and personalized care may not only enhance adherence but also facilitate early intervention and prevention strategies, ultimately improving clinical outcomes. Due to significant heterogeneity in study designs, interventions, and outcome measures among the included studies, conducting a meaningful meta-analysis may not be feasible or appropriate at this point. Meta-analyses are typically most effective when the studies included in the analysis are sufficiently homogenous to allow for meaningful comparisons and pooling of data. However, in our systematic review, the diverse nature of the studies and the variability in methodologies may limit the interpretability and reliability of results. Instead, we opted to provide a narrative synthesis of the findings, which allowed us to qualitatively summarize and contextualize the evidence from the included studies. This approach enabled us to highlight key trends, common themes, and areas of consensus or divergence among the studies, providing valuable insights into the benefits and challenges of smart technologies in DFU management. Future reviews could consider conducting a meta-analysis.

## Figures and Tables

**Table 1:** Review question (PICO).

Population	Intervention	Comparison	Outcome
Diabetic patients/Diabetic foot ulcers (DFUs)/Neuropathy	Smart insoles/footwear/Smart sensor technologies	Different groups of patients/healthy, technology, type of studies	Monitoring/prevention/care/adherence

## Data Availability

No available data.
